# Familial risks between giant cell arteritis and Takayasu arteritis and other autoimmune diseases in the population of Sweden

**DOI:** 10.1038/s41598-020-77857-7

**Published:** 2020-11-30

**Authors:** Hauke Thomsen, Xinjun Li, Kristina Sundquist, Jan Sundquist, Asta Försti, Kari Hemminki

**Affiliations:** 1 Division of Molecular Genetic Epidemiology, German Cancer Research Centre (DKFZ), 69120 Heidelberg, Germany; 2 Center for Primary Health Care Research, Lund University, Malmö, Sweden; 3 Department of Family Medicine and Community Health, Department of Population Health Science and Policy, Icahn School of Medicine at Mount Sinai, New York, NY USA; 4 Department of Functional Pathology, School of Medicine, Center for Community-Based Healthcare Research and Education (CoHRE), Shimane University, Matsue, Japan; 5 Hopp Children’s Cancer Center (KiTZ), Heidelberg, Germany; 6 Division of Pediatric Neurooncology, German Cancer Research Centre (DKFZ), German Cancer Consortium (DKTK), Heidelberg, Germany; 7 Division of Cancer Epidemiology, German Cancer Research Centre (DKFZ), 69120 Heidelberg, Germany; 8 Faculty of Medicine and Biomedical Center in Pilsen, Charles University in Prague, 30605 Pilsen, Czech Republic; 9 GeneWerk GmbH, Im Neuenheimer Feld 582, 69120 Heidelberg, Germany

**Keywords:** Risk factors, Epidemiology, Genetics research

## Abstract

Giant cell arteritis (GCA, also called temporal arteritis) is a rare and Takayasu arteritis (TA) is an even rarer autoimmune disease (AID), both of which present with inflammatory vasculitis of large and medium size arteries. The risk factors are largely undefined but disease susceptibility has been associated with human leukocyte antigen locus. Population-level familial risk is not known. In the present nation-wide study we describe familial risk for GCA and for GCA and TA with any other AID based on the Swedish hospital diagnoses up to years 2012. Family relationships were obtained from the Multigeneration Register. Familial standardized incidence ratios (SIRs) were calculated for offspring whose parents or siblings were diagnosed with GCA, TA or any other AID. The number of GCA patients in the offspring generation was 4695, compared to 209 TA patients; for both, familial patients accounted for 1% of all patients. The familial risk for GCA was 2.14, 2.40 for women and non-significant for men. GCA was associated with 10 other AIDs and TA was associated with 6 other AIDs; both shared associations with polymyalgia rheumatica and rheumatoid arthritis. The results showed that family history is a risk factor for GCA. Significant familial associations of both GCA and TA with such a number of other AIDs provide evidence for polyautoimmunity among these diseases.

## Introduction

Giant cell arteritis (GCA, also called temporal arteritis or cranial arteritis), is a systemic inflammatory vasculitis of medium and large arteries, with a possible sequelae of an ischemic optic neuropathy and irreversible or significant visual loss^1^. Left untreated, it can result in many systemic, neurologic, and vascular complications, including thoracic aortic aneurysms^2^. Although the temporal artery is most commonly involved, other arteries may also be affected. GCA is commonly associated with polymyalgia rheumatica^1^. The disease usually occurs at age over 50 years, and women are more commonly affected compared to men^2^. The prevalence in USA is estimated at 30/100,000^3^. Although the exact etiology of GCA is unknown, various genetic, environmental, and autoimmune etiologies have been hypothesized^4^. GCA is a disease of cell-mediated immunity, and the pathophysiology involves activation of adventitial dendritic cells via Toll-like receptors (TLRs) 2 and 4. These in turn activate the differentiation and recruitment of T cells, which produce interferon gamma, a key driver of vascular inflammation and hyperplasia in GCA^2^. Genetic studies have demonstrated that GCA is associated with the human leukocyte antigen (HLA) class II molecules^4^. Associations with other suggested genes include intercellular adhesion molecule (ICAM)-1, a chemokine ligand CCL5 (RANTES), and interleukin (IL) 1 receptor^4^.

Takayasu disease (TA) is another vasculitis of medium and large-sized arteries. TA progresses in two phases, in the first systemic phase, patients present with symptoms of active inflammation while the second phase is the occlusive phase, which presents with symptoms of stenosis in affected arteries. These may include claudication of the muscle groups receiving blood from the affected vessels^2^. Thoracic aortic aneurysms may be a serious complication also in TA. TA typically presents in female patients younger than 40 years. The prevalence in USA is estimated at 0.3/100,000, thus 1/100 of that of GCA^3^. The pathophysiological mechanisms in TA are similar to GCA^4^. Genetic associations for TA have been shown with HLA loci and IL12B^4^.

We were not able to find studies reporting on familial clustering of GCA or TA. Instead, there is the well-known co-morbidity of GCA with polymyalgia rheumatica; 40–60% of patients with GCA have polymyalgia rheumatica while 16–21% of patients with polymyalgia rheumatica have GCA^5^. Ulcerative colitis has been described as a co-morbidity in TA^6^.

Here we provide a detailed assessment of familial risks for GCA and TA based on Swedish hospital records. A concordant familial risk was calculated for both of these diseases and a discordant risk was calculated between these diseases and any other of 41 AIDs.

## Results

A total of 769,991 patients with any of 43 AIDs have been identified, of which 51% were received from the Inpatient Register and 49% from the Outpatient Register (see “Methods” section). By the end of the follow-up in year 2012 the offspring generation reached age 80 years. There was no age limits for the parental generation. The dataset contained a total number of 4695 GCA patients in the offspring generation (to whom risks were calculated) with a corresponding mean diagnostic age (i.e., first hospital contact) of 64.3 years. Taking their parents into account the total number of individuals resulted in 12,882 of which 28.5% were males. Overall, offspring with GCA had 52 (1.1%) first-degree relatives (parents or siblings) that were also diagnosed with GCA. The number of affected offspring for TA was 209 (mean diagnostic age 44.5 years) and the total number of patients with TA was 346. About 17.7% of all TA patients were males. Only 2 (1.0%) first-degree relatives were diagnosed with TA. The AID population in total amounted to 519,180 patients in the offspring generation of 8.517 million individuals.

### Concordant familial risks

Familial risks for GCA are shown in Table 1 for offspring whose first-degree relatives (parents or siblings) were diagnosed with GCA. The overall familial risks for GCA was 2.14 and only the female SIR of 2.40 was significant. The SIRs were practically equal when parents (2.04) of siblings (1.94) were probands. For TA the risk for offspring of affected parents was 196 (95% CI 18–721), but only based on 2 cases; data not shown. Spousal familial risks were calculated for a husband given a concordant GCA in the wife; the SIR of 1.39 was not significant. No concordant spouses with TA were found.

**Table 1:** Familial and spousal risks of concordant giant cell arteritis.

	All					Women				Men			
	O	SIR	P*	95% CI		O	SIR	95% CI		O	SIR	95% CI	
Giant-cell arteritis	52	**2.14**	**1.4 × 10**^**–4**^	**1.60**	**2.81**	38	**2.40**	**1.70**	**3.29**	14	1.67	0.91	2.80

	Parents only					Sibling only				Both parent and sibling			
	O	SIR		95% CI		O	SIR	95% CI		O	SIR	95% CI	
Giant-cell arteritis	23	**2.04**		**1.29**	**3.07**	33	**1.94**	**1.33**	**2.73**	0			

	Husbands				Wives								
	O	SIR	95% CI		O	SIR	95% CI						
Giant-cell arteritis	21	1.39	0.86	2.12	21	1.42	0.88	2.17					

*O* observed number of cases, *SIR* standardized incidence ratio, *CI* confidence interval.

Bold type: 95% CI does not include 1.00.

*P values Bonferroni-corrected; 0.00 ≤ 1.00 × 10^–180^.

### Discordant familial risks

**Figure 1:**
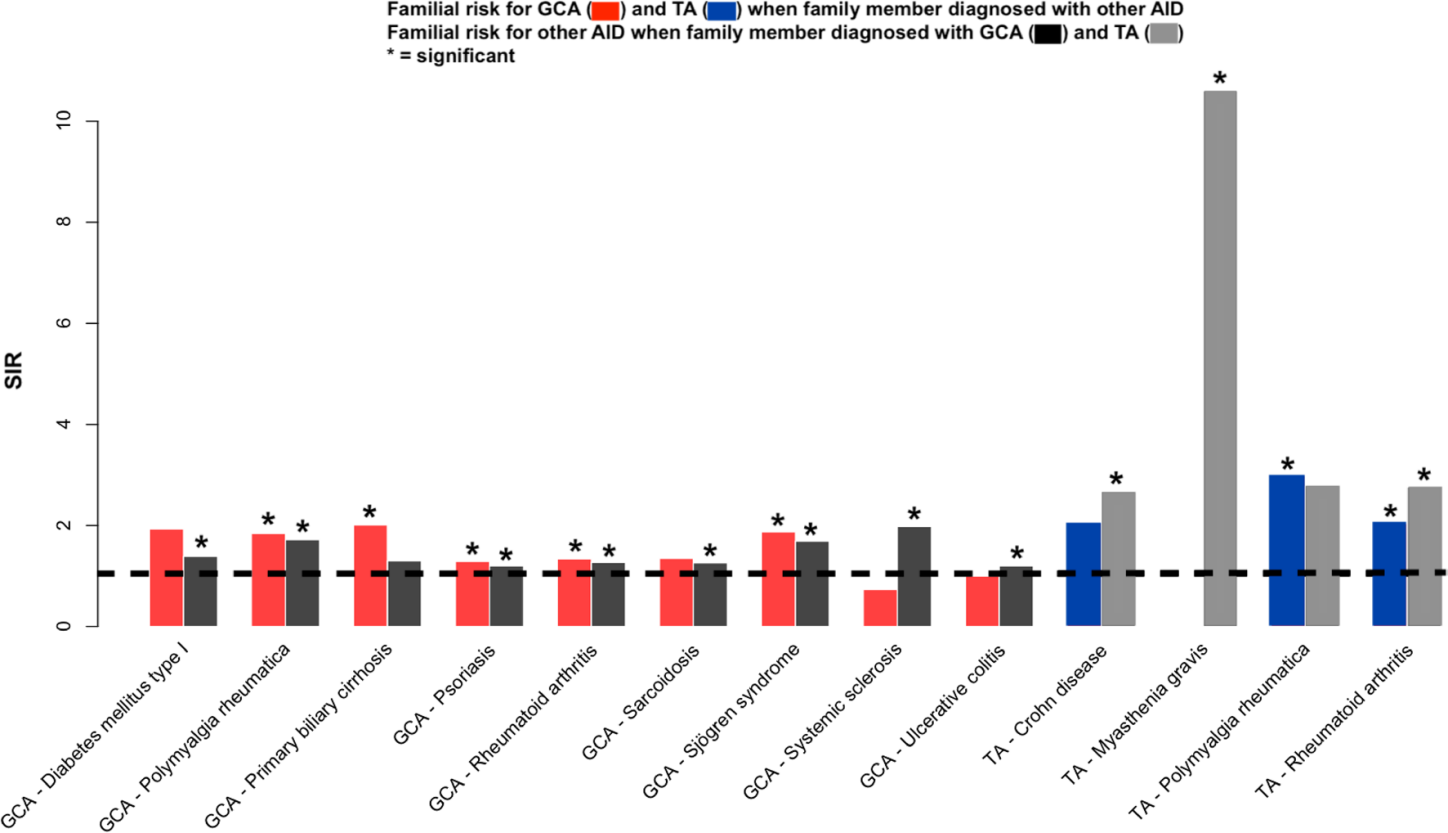
Discordant associations for giant cell arteritis (GCA) and Takayasu disease (TA) with other discordant autoimmune diseases (AIDs). Data are presented for combined sexes when at least one association was significant.

We analyzed familial risks between GCA or TA and all 41 other AIDs and the significant discordant associations are shown in Table 2. For GCA, associations with 10 AIDs were significant when considering even sex-specific results. Significant bidirectional associations (combined sexes) were noted with polymyalgia rheumatica (SIR for GCA 1.84, reverse 1.72), psoriasis (1.29/1.20), rheumatoid arthritis (1.34/1.27) and Sjögren syndrome (1.87/1.69). Considering the female excess in overall case numbers, many more female associations were consequently significant compared to male associations. TA showed a significant association with 6 AIDs, of which that with rheumatoid arthritis (2.04/2.76) was bidirectional. Both GCA and TA were associated with polymyalgia rheumatica and rheumatoid arthritis. The significant discordant associations are summarized in Fig. 1. Only one family had members diagnosed with both GCA and TA (SIR for GCA 2.28, 0.0–8.92; SIR for TA 1.13, 0.0–4.42).

**Table 2:** Discordant familial risks for giant cell arteritis and Takayasu arteritis.

Subtypes of AID in offspring	Family history of AID	Both Genders				Men				Women			
		Obs	SIR	95% CI		Obs	SIR	95% CI		Obs	SIR	95% CI	
Giant-cell arteritis	Diabetes mellitus type I	6	1.93	0.69	3.78	2	1.95	0.18	5.59	4	1.92	0.50	4.26
**Diabetes mellitus type I**	**Giant-cell arteritis**	**65**	**1.39**	**1.07**	**1.74**	26	1.05	0.69	1.49	**39**	**1.76**	**1.25**	**2.36**
Giant-cell arteritis	Pemphigoid	14	1.23	0.67	1.95	5	1.30	0.41	2.70	9	1.19	0.54	2.09
**Pemphigoid**	**Giant-cell arteritis**	8	1.55	0.66	2.82	1	0.41	0.00	1.60	**7**	**2.59**	**1.03**	**4.86**
Giant-cell arteritis	Polymyalgia rheumatica	**144**	**1.84**	**1.55**	**2.15**	**47**	**1.72**	**1.27**	**2.25**	**97**	**1.90**	**1.54**	**2.29**
**Polymyalgia rheumatica**	**Giant-cell arteritis**	**67**	**1.72**	**1.33**	**2.16**	**26**	**1.57**	**1.02**	**2.23**	**41**	**1.84**	**1.32**	**2.44**
Giant-cell arteritis	Primary biliary cirrhosis	**15**	**2.01**	**1.12**	**3.16**	6	2.40	0.86	4.71	9	1.81	0.82	3.19
**Primary biliary cirrhosis**	**Giant-cell arteritis**	11	1.30	0.64	2.18	4	2.80	0.73	6.22	7	0.99	0.39	1.86
Giant-cell arteritis	Psoriasis	**190**	**1.29**	**1.11**	**1.48**	64	1.26	0.97	1.59	**126**	**1.30**	**1.08**	**1.54**
**Psoriasis**	**Giant-cell arteritis**	**463**	**1.20**	**1.09**	**1.31**	**235**	**1.25**	**1.09**	**1.41**	**228**	**1.15**	**1.00**	**1.30**
Giant-cell arteritis	Rheumatoid arthritis	**270**	**1.34**	**1.19**	**1.51**	**91**	**1.31**	**1.06**	**1.60**	**179**	**1.36**	**1.16**	**1.56**
**Rheumatoid arthritis**	**Giant-cell arteritis**	**271**	**1.27**	**1.12**	**1.42**	**90**	**1.38**	**1.11**	**1.68**	**181**	**1.22**	**1.05**	**1.40**
Giant-cell arteritis	Sarcoidosis	41	1.35	0.97	1.80	**21**	**1.98**	**1.23**	**2.92**	20	1.01	0.62	1.50
**Sarcoidosis**	**Giant-cell arteritis**	**84**	**1.26**	**1.00**	**1.54**	37	0.94	0.66	1.27	**47**	**1.71**	**1.26**	**2.23**
Giant-cell arteritis	Sjögren syndrome	**26**	**1.87**	**1.22**	**2.66**	**11**	**2.25**	**1.12**	**3.77**	15	1.67	0.93	2.62
**Sjögren syndrome**	**Giant-cell arteritis**	**48**	**1.69**	**1.24**	**2.20**	4	1.50	0.39	3.34	**44**	**1.71**	**1.24**	**2.25**
Giant-cell arteritis	Systemic sclerosis	4	0.73	0.19	1.61	1	0.55	0.00	2.14	3	0.82	0.15	2.00
**Systemic sclerosis**	**Giant-cell arteritis**	**16**	**1.98**	**1.13**	**3.07**	2	1.10	0.10	3.16	**14**	**2.24**	**1.22**	**3.56**
Giant-cell arteritis	Ulcerative colitis	69	1.00	0.78	1.25	19	0.81	0.49	1.22	50	1.10	0.82	1.42
**Ulcerative colitis**	**Giant-cell arteritis**	**232**	**1.20**	**1.05**	**1.36**	116	1.13	0.93	1.34	**116**	**1.29**	**1.06**	**1.53**
Takayasu arteritis	Crohn disease	4	2.05	0.53	4.54					4	2.62	0.68	5.81
**Crohn disease**	**Takayasu arteritis**	**7**	**2.67**	**1.06**	**5.02**	**6**	**4.61**	**1.66**	**9.03**	1	0.76	0.00	2.97
Takayasu arteritis	Graves	6	1.91	0.69	3.75					**6**	**2.51**	**0.90**	**4.93**
**Graves**	**Takayasu arteritis**	3	1.05	0.20	2.58					3	1.26	0.24	3.08
Takayasu arteritis	Hashimoto thyroiditis	5	2.57	0.81	5.32	1	2.30	0.00	9.01	4	2.65	0.69	5.89
**Hashimoto thyroiditis**	**Takayasu arteritis**	6	2.57	0.93	5.05					**6**	**3.10**	**1.11**	**6.07**
Takayasu arteritis	Myasthenia gravis												
**Myasthenia gravis**	**Takayasu arteritis**	**2**	**10.60**	**1.00**	**30.38**	1	13.51	0.01	52.98	1	8.72	0.00	34.18
Takayasu arteritis	Polymyalgia rheumatica	**7**	**2.98**	**1.18**	**5.60**	1	1.46	0.00	5.73	**6**	**3.61**	**1.30**	**7.07**
**Polymyalgia rheumatica**	**Takayasu arteritis**	2	2.79	0.26	7.99	1	3.39	0.00	13.31	1	2.36	0.00	9.27
Takayasu arteritis	Rheumatoid arthritis	**15**	**2.04**	**1.14**	**3.20**	2	1.04	0.10	2.98	**13**	**2.39**	**1.27**	**3.87**
**Rheumatoid arthritis**	**Takayasu arteritis**	**12**	**2.76**	**1.42**	**4.54**	**4**	**3.01**	**0.78**	**6.69**	**8**	**2.64**	**1.13**	**4.79**

Bold values indicate 95% CI does not include 1.00.

## Discussion

To our knowledge this is the first family study on GCA and TA, which were earlier falsely considered to be the same disease of inflammatory vasculitis of large and medium size arteries, in spite of the different age distributions^2^. The present results showed that the familial clustering of GCA and TA is rare, as only 1% of the patients had a concordant family history. The familial risk for GCA was 2.14 and it was only significant for women (2.40), which is noteworthy considering the sex difference in incidence. In diseases with large sex differences, stronger familial effect may be found in the gender of lower background incidence, which is referred to as “the Carter effect”, described for pyloric stenosis^7,8^.

There was no difference in risk when the analysis was done between offspring and parent or between siblings. A higher sibling risk might indicate recessive inheritance or influence of environmental sharing in the childhood. Thus, the present results gave no indication for such contributions. Lacking spouse correlation suggested that even adult environmental sharing played no major role for GCA susceptibility. The familial risk for TA was very high (SIR 196) but as it was based on only 2 cases, it needs to be confirmed with larger case numbers.

In spite of the rare concordant familial clustering, there were 10 discordant associations for GCA and 6 for TA. For GCA, SIRs were about 1.8 with polymyalgia rheumatic and 2.0 with primary biliary cirrhosis, i.e., not much lower than the concordant SIR, which suggests extensive genetic (or environmental) sharing between GCA and these AIDs. Risks were also high for associations with Sjögren syndrome and systemic sclerosis. For TA only rheumatoid arthritis showed bidirectional associations but these were nominally higher than those with GCA. The two vasculitis were both associated with rheumatoid arthritis and polymyalgia rheumatica; the latter association for GCA was not unexpected because of the known co-morbidity. Ulcerative colitis has been described as a co-morbidity for TA, and we found an association of ulcerative colitis in families of GCA^6^. In spite of the shared clinical features and pathophysiology, GCA and TA were not associated with each other which is probably due to the rarity of familial clustering of these diseases; only one family was found with a patients with GCA and another with TA^4^. Polyautoimmunity (i.e. presentation of more than one AID in the same individual) is a common feature of many AIDs, and often include thyroid AIDs and type 1 diabetes, both of which manifested also in the present study. The genetic basis of such pleiotropy is partially understood (1, 4); genome-wide association studies have shown that low-penetrance loci are extensively shared between AIDs^9–11^. In a study of pediatric AIDs over 70% of the significant genetic loci were shared by at least 3 AIDs^12^. The HLA locus is involved in many AIDs, including GCA and TA^4^. The present data provide strong evidence for polyautoimmunity for GCA and TA, and should call for further characterization of the shared genetic loci.

Limitations of the study are inherent to the rarity of these diseases, particularly TA with in total 346 nationally identified patients. In addition, drastically different age distributions of these vasculitis complicate comparisons between the two. Another problem is also inherent to these diseases, i.e., the co-morbidity of GCA with polymyalgia rheumatica. Our sampling strategy defined inclusion of patients by the first AID diagnosis only. Thus, patients were defined as polymyalgia rheumatica patients even though they might have been diagnosed shortly afterwards with GCA.

In conclusion, the present study provided novel familial risk estimates for GCA and identified discordant associations for GCA with 10 other AIDs and for TA with 6 other AIDs. Significant familial associations with so many other AIDs provide evidence for polyautoimmunity among these diseases.

## Methods

Methods used were in principle similar to those in our previous family studies on cancer and autoimmune diseases^13–15^. We identified AID patients from the Swedish Hospital Discharge Register (years 1964 through 2012, full national coverage from 1986 onwards) and the Outpatient Register (2001 through 2012). Only the first AID diagnosis was included. Various revisions of the International Classification of Diseases (ICD) codes were used for identification of AIDs as described elsewhere^14^. Family relationships were obtained from the Multigeneration Register, containing the Swedish population in families and covering parental generations for a century^16^. As family members, only first-degree relatives of offspring-parent pairs and siblings were considered; ‘the offspring generation’ was born after 1931 and siblings could be defined only in this generation; ‘the parental generation’ was born any time earlier. Information from the registers was linked at the individual level via the national 10-digit civic registration number. In the linked dataset, civic registration numbers were replaced with serial numbers to ensure the anonymity of all individuals. Spouses were identified through the first common child.

Standardized incidence ratios (SIRs) were calculated for the offspring generation as the ratio of observed to expected number of cases. The expected numbers were calculated for all individuals without a first-degree family history of a specific AID, and the rates were standardized by 5-year-age, gender, period (5 years group), socioeconomic status and residential area. The 95% confidence interval (95% CI) of the SIR was calculated assuming a Poisson distribution. Separate SIRs were calculated for offspring when only parents or only siblings were probands, i.e., they were diagnosed with concordant AID^15^. In analysis of discordant AIDs bidirectional (i.e., GCA-AIDx and AIDx-GCA) associations were considered.

### Ethics

The guidelines of the Helsinki Declaration were followed. The study was approved by the Regional Ethical Review Board in Lund—the so called “Regionala Etikprövningsnämnden i Lund”. The study was conducted in accordance with the approved guidelines with explicit statement that no informed consent was required, because the study is a national register-based study on anonymous personal data. Therefore, the ethical committee “Regionala Etikprövningsnämnden i Lund” waived the need of informed consent.
